# Proving the proverbial gadfly: situating the historical and racial context of Southern medical works by Mary Louise Marshall

**DOI:** 10.5195/jmla.2021.1261

**Published:** 2021-10-01

**Authors:** Aidy Weeks

**Affiliations:** 1 aidy.weeks@gmail.com, https://twitter.com/wonderbrarian, University of Nevada, Las Vegas, Las Vegas, NV

**Keywords:** history of health sciences librarianship, historical revisionism, whiteness in LIS, critical race theory, critical librarianship, library leaders, MLA, JMLA, integration

## Abstract

Health sciences librarianship has historically benefited from avoiding critical conversations around the role of race in the profession, reflected through a select few number of articles on the topic. The purpose of this study was to add to this body of literature and apply a critical librarianship framework on the early scholarly record of health sciences librarianship and the legacy of integration within the Medical Library Association (MLA). Three Southern medical works and the integration views of Mary Louise Marshall, the longest-serving president of MLA from 1941 to 1946, were thematically and textually analyzed to redress the profession's long-standing legacy with Whiteness and Black, Indigenous, and People of Color (BIPOC) representation. In reframing the historic past of MLA both through Marshall's works and her views, the goal is to acknowledge ways in which the profession has impeded progress and present steps to remedy appropriate outreach for the future.

## INTRODUCTION

It has happened—the visible dissolution of the colorblind boundaries by which health sciences librarianship frames its discourse. Influential works such as those on diversity and inclusion, the arrival of #critlib or critical librarianship in health sciences scholarship, and open condemnation on the brutalization of institutionalized racism and state-sanctioned violence against Black and Brown individuals are important narratives threading their way in a technical and competencies-focused field [1–3]. Efforts to address racism and social justice issues by the *Journal of the Medical Library Association* (*JMLA*) have led to the outreach of Black, Indigenous, and People of Color (BIPOC) health sciences librarians to address representation in the scholarly pipeline. BIPOC health sciences librarians are being asked for their thoughts, opinions, and ideas with genuine interest. Such inquiry exchange not only signals that the profession is listening but also serves as the impetus for this paper's goal to address how the scholarly landscape reckons with a problematic past in order to welcome a socially conscious future.

## BACKGROUND

This paper is derived from an opportunity to provide feedback to past and current editors of *JMLA*. In July 2020, a *JMLA* editor made an initial request for feedback from leaders of the African American Medical Librarians Alliance (AAMLA) and Latinx Caucuses. *JMLA* editors received input for areas in which the publication could improve within its editorial board and peer reviewers and through targeted outreach of BIPOC librarians interested in scholarly publishing [4].

The author of this paper shared feedback for the consideration of the publication's history, inherent biases within the editorial process, and the review of problematic articles that contrast against the new goals of the journal's editorial body. The author felt that in doing the work to improve future efforts, reflecting on the past was equally important. This feedback led to follow-up questions on how this was possible, and in response to that inquiry, this paper seeks to examine a sampling of the *JMLA* historical scholarly record and by extension share recommendations in which the publication can build a space for current and future BIPOC authors.

In order to critically examine this topic, the author applied a critical librarianship theoretical framework focused on the historical and racial context of articles found in *JMLA* [5]. More specifically, this examination reviewed older works found within the predecessor of *JMLA*, the *Bulletin of the Medical Library Association* (*BMLA*), by Mary Louise Marshall, the longest-serving president of MLA. Three key works, along with Marshall's views on integration, were analyzed in order to contextualize the journal's historical record on addressing race within the profession. Through a historical examination of racial biases within scholarship and the profession, this paper invites critical conversations of discourse in the future.

This paper posits the following research question:

What evidence exists of *JMLA*'s history with problematic articles? How should we reflect on the past in order to inform scholarly diversity, equity, and inclusion goals for the future?

## THEORETICAL FRAMEWORK

In order to examine the legacy of Mary Louise Marshall as both a leader and scholar, the author incorporated Historical Revisionism theory [6], Whiteness as Property from Critical Race Theory (CRT) [7], and Whiteness in LIS theory [8–9] into a critical librarianship framework. In considering the concept of time, Dabrinski writes, “critical librarianship must grapple with librarianship's relationship to time, to a past accumulation that represents an ordering of only certain kinds of things, reflective of only dominant modes of seeing and making the world” [10]. This framework, as applied, illuminates facets of the dominant narrative within the health sciences library profession that were disregarded.

Library practitioners and theorists examine librarianship through a critical lens. From the 1970s through the 1990s, a proliferation of library ethnic caucuses, as well as affinity group round tables, were a testament to the awareness of and advocacy for critical perspectives outside the dominant narratives within the field [11]. In 1991, Buschman and Carbone aimed to connect critical inquiry and librarianship to the New Sociology of Education theoretical framework, whereby “the mark of this field of study has been the insistence that social institutions and those who work in them cannot be studied apart from their social, political, and economic environments” [12]. Buschman and Carbone proposed that in order for librarianship to effectively examine library information studies, it must be done through a lens of inquiry that acknowledges its role with “power, race, class, and gender in late Western capitalist democracies” [13].

In 2019, Barr-Walker and Sharifi provided a primer on how this framework could be applied to health sciences librarianship, detailing specific examples on how critical librarianship can be integrated into the profession's praxis from “technical services and cataloguing” to a broader scope of “libraries and librarianship” [14]. They concluded by stating, “acknowledging that health sciences libraries and library workers are not neutral is the first step in addressing broader issues in our organizations and profession. Health sciences librarians can critically evaluate our services and spaces and advocate for action to address inequities in our libraries, in our professional associations, and in the broader field” [15]. This paper continues their call to action by addressing how Marshall's racist ideologies and the publishing of her views reflect the non-neutral reality of the health sciences librarianship scholarly record. To date, this is the first paper to attempt this approach.

For this work, the author sought clarity on how history and racism played a role in the narratives found within the annals of the MLA's flagship publication. In examining the history of library and information studies literature, Velez and Villa-Nicholas point to the only two articles written explicitly about racism in the health sciences librarianship field [16]. These two articles written by Carolyn Lipscomb, “Race and Librarianship: Part I” (2004) and “Race and Librarianship: Part II” (2005), proved to be insightful works in understanding how anti-Blackness sentiment was present during the early days of the association [17–18].

### Historical Revisionism

When Theodor Mommsen, the great German historian of the nineteenth century, pointed out that each generation interprets history in the light of its own experience … he was merely stating what must be obvious to everyone: the same facts may mean quite different things to different people. Since librarianship is just now arriving at the point of looking back at itself historically, it is not surprising that some library historians are suggesting a new interpretation of past events [19].

Estelle Brodman, former president of MLA and editor of *BMLA*, wrote those words forty years ago in 1980 [20]. Brodman used Historical Revisionism to explain the early history of MLA as it pertained to gender dynamics and the influx of women into medical librarianship. The key to this theory is not to *revise* history as stated in its name, but to *reinterpret* history using a theory that best supports the evaluation of a particular issue from the past.

Brodman's framework of Historical Revisionism, when applied to medical librarianship, grounds a newer theoretical framework that focuses on race and how it relates to narratives and legacy within the profession. Coincidentally, Marshall's oral history was the subject for this original framework as Brodman was her interviewer [21].

### Whiteness as Property in Critical Race Theory

To anchor this new theoretical framework with respect to the narrative of race in Marshall's works, the author applied the Whiteness as Property model from CRT, coined by Cheryl I. Harris. Harris used this legal model to explain the interconnected nature between slavery, Whites, and property and how “the hyperexploitation of black labor was accomplished by treating black people themselves as objects of property. Race and property were thus conflated by establishing a form of property contingent on race” [22]. In this examination of medical librarianship, Marshall instituted ownership of enslaved narratives by invoking her authority and expertise as a librarian and historian. In essence, Marshall becomes an owner-by-proxy in the retelling of enslaved narratives she shared with the profession both as an orator and an author. By incorporating this theory, a clearer picture is found in how White-narratives-as-property contributed to the romanticizing and dismissiveness for the brutal history of slavery in order to allow palatable consumption by members of the profession.

### Whiteness in LIS Theory

In order to understand how Marshall's works and legacy are situated within the profession, it is important to consider the ways a White-majority profession can overlook her works and uphold an overall positive legacy. Evidence for why this model should be applied is found in both the most recent MLA demographic survey, wherein 73% of survey respondents self-identified as White and within the literature, which has described the historical trend of remaining a majority White profession [23–24]. Two foundational works within the library and information studies (LIS) literature that helped to contextualize the profession's relationship to Whiteness and color-blindness on race are Espinal and Honma. In 2001, Espinal applied “whiteness studies” as a conceptual framework for addressing inequality issues within the profession, noting, “unless we identify and name it, many of the problems that plague us collectively and as individual librarians of color will continue” [25]. Espinal wrote, “currently there are crises and problems in librarianship that have been articulated in terms of the profession's response to diversity … These crises and problems stem from the field's very constitution as a white profession and cannot be solved or even tackled until the facts of whiteness in librarianship and libraries have been exposed in a systematic way” [26]. Nearly twenty years ago, Espinal understood that achieving progress in diversity, equity, and inclusion within librarianship required acknowledgement that the profession's Whiteness was a formidable barrier to those goals.

In 2005, Honma discussed the “white racial normativity” so prevalent in LIS that it permeated and obscured the need for critical discussions on race and librarianship [27]. Honma goes on to state that “the identification of whiteness and its structuralizing principles is necessary in order to combat its invisibility and normative effects” [28]. More recently, library scholars continue addressing the longstanding issue of how Whiteness—the power and privilege embodied therein—serves to undermine librarianship at large from its progress toward diversity, equity, and inclusion of BIPOC library workers [29–31]. Though the discussion of Whiteness exists within academic librarian scholarship, rarely has it filtered into health sciences librarianship discourse. Only in recent years has progress been made in acknowledging Whiteness as a problem within the profession [32]; however, mentioning that it is an issue still requires action. As Whiteness in LIS continues to be discussed, it is imperative that it continues to be called out and addressed if critical discourse about barriers and exclusionary practices by the profession takes place. A grounded historical review on Whiteness in LIS in health sciences librarianship provides undeniable evidence that it is real, has always existed, and carries significant ripple effects in how the profession treats BIPOC library workers.

**Figure 1:**
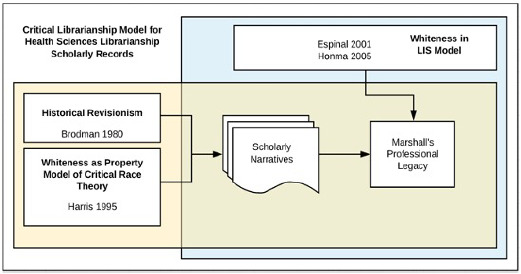
Critical librarianship model for historical health sciences librarianship scholarly records

Distinct parts of the outlined theoretical framework aim to address Marshall's works and her legacy separately; however, an overlap exists in which both Historical Revisionism and Whiteness as Property model can be used to address Marshall's legacy while the Whiteness in LIS theory can be used to address *JMLA*'s historical scholarly record (Figure 1).

## METHODS

The articles in this review were written during the tenure of *BMLA* and range from 1938 to 1957. All articles were solely authored by Mary Louise Marshall. The author searched past issues of *JMLA* within PubMed Central to locate any instances within the scholarly record that alluded to racism and met racial criteria. Originally, the author scanned each issue from 1898 to 1938 until landing on “Plantation Medicine” by Mary Louise Marshall [33]. In cross-referencing Mary Louise Marshall's name in Lipscomb's “Race and Librarianship: Part I,” the author decided to focus on the scholarly contributions and views of Marshall [34].

**Figure 2:**
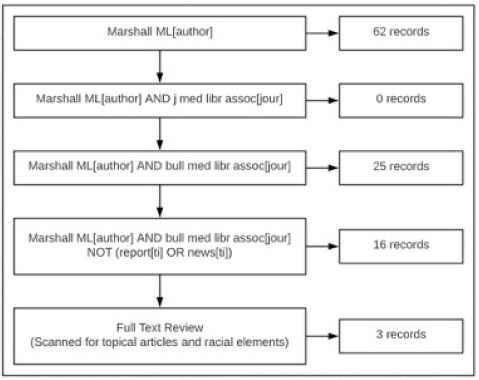
Literature search flow chart

The author reviewed all writings by Marshall and any contributions that mentioned her involvement within MLA. There were sixty-two records for “Marshall ML” with the author tag “[author]” in PubMed. The search was revised to include any citations written by Mary Louise Marshall in *JMLA*. That search returned no records. The search was again revised to look for any citations written in *BMLA*, which returned twenty-five records. Any contributions related to MLA reports or news items were excluded. That search returned sixteen records. The author then conducted a full-text scan of all works and selected the articles that were based on Southern medical history and met racial criteria. This reduced the total number of records to three. All three have been included in this study (Figure 2).

### Qualitative Analysis Approach

The author then completed an initial read of each text, rescanned each text, and pulled passages that related to racial criteria, using terms like “slave,” “slavery,” “black,” “white,” “negro,” and “colored” and placed those phrases in a Google Doc. The author then bolded terms within each passage to assist with future coding and provided a short annotation or commentary for each one. After conducting an initial scan with annotation, the author began to outline topical areas to assist in the critical review. Each phrase found from the scanned articles, along with its page number and annotation, was then collated to the topical area with a tag for the article title and placed into Google Sheets.

## RESULTS

Three articles relevant for this critical review ranged from 1938 to 1957. They are listed in chronological order: “Plantation Medicine” (1938), “Medicine in the Confederacy” (1942), and “Nurse Heroines of the Confederacy” (1957).

## MARY LOUISE MARSHALL

### Who was Mary Louise Marshall?

Mary Louise Marshall was an influential figure during the early days of MLA. She held numerous leadership roles including chairman of the Membership Committee (1928–1929); an editorial committee member for *BMLA* (1929); “News from the Libraries” editor (1929); treasurer (1931); Executive Committee member (1938); and president (1941) [35–40]. Marshall would go on to be the longest-serving president of MLA (1941–1946), was instrumental in helping to establish the National Library of Medicine, the successor to the Army Medical Library, and worked on two editions of the seminal *Handbook of Medical Library Practice* [41]. She was also the recipient of the Marcia C. Noyes Award for her contributions to the enhancement of medical librarianship and served a combined thirty-nine years as the medical librarian for the Orleans Parish Medical Society and Tulane University Medical School Library [42]. In addition, Marshall was also one of the founders of the Southern Chapter of the Medical Library Association [43].

### In Favor of Whiteness and Against Integration

Marshall also played a significant role in obstructing the future integration of MLA. In “Race and Librarianship: Part I,” Lipscomb described concerns in 1939 by MLA leadership related to the admittance of Howard University and Meharry Medical College medical libraries, both historically Black institutions. She described how Janet Doe presented two questions to the Executive Committee regarding Meharry's medical library admittance: the first whether or not to admit them and the second if “other negro libraries be advised of our change of policy?”[44]. In both instances, Marshall, chairman of the Executive Committee, abstained from voting. For Marshall, her conviction for not voting was emblematic of the hegemonic nature of Whiteness in LIS. As described in her own words:

As a scientific body there is of course no reason for the exclusion of negro library members. On the other hand one of the principal advantages of our Association, —I might even say its greatest advantage, has been the opportunity which has been offered for close acquaintance with others in our field, and the amalgamation of our whole group … With my head I know this is a wrong attitude, and with my heart I regret it from the bottom of my heart, but I truly believe a serious social problem will be created for our meetings if negro librarians come to our meetings, and become a part of our group [45].

To admit Black institutions and Black librarians into MLA meant for Marshall that it would disrupt the close-knit and exclusively White community of professionals that was “its greatest advantage.” Furthermore, her use of the conjunction “but” contradicts her previous statement and dismisses any genuine sense of guilt or shame in her opinion. Even with Marshall's first abstention, the initial vote by Executive Committee members was in favor of admitting both Howard's and Meharry's medical libraries. However, her second abstention tied the vote for notifying other Black medical libraries that the “code” as noted by Lipscomb was now changed. Essentially, integration of MLA took place with the admittance of two medical libraries and no intent in making this known for others to join. It may be pointed out that this makes sense since a third and fourth medical school at a Historically Black College or University (HBCU) was not founded until 1966, with Charles R. Drew University of Medicine and Science and 1975, with Morehouse School of Medicine [46–47]. However, in 1938, a year prior to the vote for integration, Numa P. G. Adams, dean for the School of Medicine at Howard University, described how medical libraries existed in Black hospitals, though not always in the best conditions as their White counterparts [48]. It can be surmised that Dr. Adams's assertion for high-quality medical libraries in Black hospitals equal to those at White hospitals and medical schools was meant to subvertly advocate for integration. And it is to be noted that medical societies and academic and hospital libraries made up the early membership of MLA. Marshall's political decision-making at the highest levels of leadership hallmarks how Whiteness within health sciences librarianship remained dominant.

Though Lipscomb gives Marshall credit for supporting the Executive Committee in approving Meharry Medical College and Howard University and writing letters defending the decision to members, she falls short in other critical aspects of Marshall's decision-making. Marshall not only prevented transparency in this policy change, eliminating a chance for admittance of other Black institutions, but Lipscomb goes on to remark that one of the reasons Marshall approved was “the unlikely case that similar libraries would become eligible” [49]. In Marshall's view, Black medical libraries were inferior to the standards of MLA. Her tokenism of the issue proved to be a superficial effort toward the goal of integration, while withholding the ability to promote actual change. For both institutions, Lipscomb notes, their struggle to integrate took several years and three separate discussions by the Executive Committee. For these two institutions, being admitted into MLA was only a partial victory.

## SOUTHERN WORKS

### A Timeline of Her Works

On May 25, 1937, in Richmond, Virginia, the former capital of the Confederacy, Marshall, then executive secretary of MLA, stood before members of the association and read “Plantation Medicine,” based on her findings of old manuscripts on plantation life in Louisiana [50]. It would go on to paint a romanticized picture of life on the plantation for those who lived and labored there. For Marshall, this was an opportunity to enlighten the audience with information about what it was like to practice medicine in the Deep South.

Five years later, in May 1942, in New Orleans, Louisiana, Marshall, now president of MLA, stood once more before members of MLA and read “Medicine in the Confederacy” as a way to parallel the similarities between the Second World War and the crisis of a scarcity of resources that the nation faced [51]. In the backdrop of the burgeoning boycott and civil rights movements of the South, Marshall published her last article in her trilogy on medicine in the Deep South with “Nurse Heroines in the Confederacy” in 1957 [52].

### On Race and Medicine

Marshall was fascinated by Southern history and medical practice. The discovery and review of old manuscripts that formed her work “Plantation Medicine” was a “most absorbing subject” [53]. As a librarian and historian, she noted that there was limited information available on the health and care of enslaved populations and therefore felt she had an obligation to fill this gap in the literature.. However, her views on the subject matter and ascribing race as a biological factor rather than a political and social factor no longer lend to an accurate understanding of how race, genetics, and medicine perpetuate racial essentialism.

Marshall's review of the physical and psychological ailments of enslaved men and women reflect scholarly views prominent during the nineteenth and early twentieth century. These include attributing common illnesses afflicting the enslaved population specifically to West African ancestry rather than health disparities derived from maltreatment and violence, and observing that pregnant enslaved women were provided with the utmost of care, when in fact they were subject to physical punishment by overseers and brutal postnatal medical experimentation by plantation doctors [54–55]. Also, the falsehood that malingering or pretending to be ill was easily remedied through nonviolent means such as trickery, when in fact plantation owners and overseers would often use violence to “prove” someone falsely enacted an illness, even if the illness was legitimate [56]. Marshall's generalization that Southern physicians provided amicable care veiled a harsh truth that they benefited from a steady supply of Black bodies to haphazardly experiment on and exploit for the advancement of early medical education [57–58].

Marshall's plea for “an authoritative and much-needed text on negro medicine” is premised with the belief that Black people were inherently susceptible to diseases separate from White people [59]. Indeed, it has been well documented that the susceptibility for diseases and chronic illnesses among the enslaved were not due to race as a biological factor but by the social structures of enslavement, oppression, and generational trauma [60].

### On White-washing and Romanticizing the Black Enslaved Experience

Of all three works, “Plantation Medicine” demonstrated Marshall's talent for spinning idyllic narratives of enslaved life in the South. She extolled positive attitudes found in manuals that described the maintenance and care of the enslaved, such as being “kind,” “considerate,” and “attentive” to ensure their “happiness” [61]. She described how plantation life functioned through positive interpersonal dynamics between both races, “as succeeding generations of black families were born on a plantation … in a community with succeeding generations of a white family, there developed a patriarchal system, characterized by mutual affection” built on “mutual regard and loyalty” [62]. For those who questioned such faulty assertions, Marshall alluded to all “the records … full of evidence of this appreciation of values … constantly emphasized in instructions to overseers, in diaries, plantation records, and even in doctors' bills” [63]. Though Marshall used these records to reinforce these “truths,” in actuality this was an example of how the enslaved experience was legitimized through the documentation and narratives by Whites.

Marshall ascribed the more positive aspects of plantation life and that of the mistress figure through the anecdotal evidence left by temporary observers, or travelers, whose fleeting witness of plantation life proved useful in eulogizing what it was like to live there. She writes, “the mistress of the plantation or in her absence the wife of the overseer was usually responsible for direction of those in charge … travelers often comment on the devotion of these ladies to the care of their charges and of the endless calls on their attention and personal help. Reference to this is made in the recently popular Gone with the Wind” and “contemporary descriptions of travelers comment on the happiness and content of the children on the plantations visited” [64].

Marshall's bias toward picturesque Southern hospitality paints a false image of the enslaved experience, as oftentimes the mistress of the plantation as much as the plantation owner was an actor to the cruel nature suffered by the enslaved. Through previously published first-hand accounts like *Twelve Years a Slave* (1853) by Solomon Northup, a freedman who was kidnapped then enslaved on a Louisiana plantation and endured and witnessed violent abuse by his owners, such depictions of perceived benevolent care quickly dissipate [65]. In truth, the sole purpose of providing care was to ensure that the enslaved were healthy enough to continue their labor in the fields. The inherent motivation for care was always economical in nature. Later works of literature on slavery and medicine document a clearer, more brutal picture of slave life and the negative racial attitudes that affected the care of those enslaved on Southern plantations [66–69].

By presenting these manuscripts in her own words, Marshall reinforced the Whiteness in Property model, effectively owning the narratives in the retelling of the Black enslaved experience. As an owner-by-proxy to these narratives, she authoritatively framed their livelihood and care as positive experiences, erasing the cruelty and commodification of their existence on the plantation. Not only were the enslaved literal property during the time frame that these manuscripts were written, but Marshall extended figurative ownership through her scholarly recounting of these records.

### On White Superiority

Marshall's views on hierarchical racial structures are most apparent in “Plantation Medicine.” She affirms the misconception of slavery as “a stage of social progress” that “emerge[s] wherever social units of *unlike order* or capacity are brought into continued competitive contact in the struggle for existence” [emphasis added] [70]. For Marshall, Blacks and Whites were not viewed equally. In her words, part of the issue with emancipation was due to “the social state which must ensue on freeing such *hordes of blacks* at one time” [emphasis added], invoking a visceral image of overwhelming chaos and conveying to readers a sense of fear-mongering that was prominent during the Reconstruction era after the American Civil War [71–72]. What is most striking in this description is the timing; Marshall published these words in 1938 and abstained on the integration vote just one year later. Though Marshall ascribed this depiction to a specific time period well before MLA's discussions on integration, her written words describing clear social hierarchies between Blacks and Whites are self-evident of her sentiments on White superiority and Black inferiority. As previously noted, it was Marshall who shared concerns about racial mixing within MLA. And it was Marshall who believed that Black libraries had a low likelihood of meeting MLA's standards for membership.

In further examining Marshall's views on the value of the Black individual, she remarks on modern-day plantation medicine and shares an anecdote about a Black servant who continued to receive care from his former owner. For Marshall, there was a limit to what a plantation owner should pay when treating the ailments of the formerly enslaved. She writes:

This ofttimes extends to unnecessary expense to afford a faithful worker satisfaction. The author was recently told of the mistress of a plantation who paid a dentist to put two gold crowns on a servant's teeth, because he was old and ill, and had always wanted two gold teeth. The doctor's bill on this plantation is sometimes forty dollars per month but is accepted as legitimate expense [73].

For Marshall, two gold crowns in exchange for a lifetime of brutality and dehumanization was too costly a transaction, even though plantation owners profited immensely from Black labor during and after the dissolution of slavery. The possibility of former-slaveholding families being able to afford such care was very probable since many of those families financially recovered after the second and third generations [74].

### On the Use of the Phrase “War Between the States” and Sympathy for the Lost Cause

In all three works, Marshall adopted the phrase “War Between the States,” a seemingly innocuous word choice used for referring to the American Civil War. Etymological review of the phrase revealed that this word choice was not accidental but preferential to those who sympathized with the Lost Cause of the Confederacy. First seen within the titles of works by prominent Southern men, including former Confederate soldiers, in the 1860s, the phrase was associated with the Lost Cause narrative that focused on the Confederacy's plight in upholding state's rights rather than the institution of slavery [75]. Toward the late 1890s, it was later adopted by prominent Southern organizations like the United Confederate Veterans and the United Daughters of the Confederacy (UDC) [76]. In 1911, the UCD later campaigned to change the name from “Civil War” to “War Between the States” by petitioning Congress and failed [77]. Shortly after the end of the American Civil War and into the early twentieth century, this phrase grew in popularity. According to Google's Ngram, this phrase had an exponential rise in popularity since about 1930 and peaked in 1940, corresponding to the date range of Marshall's first work, “Plantation Medicine” [78].

Marshall mentions this phrase twice in her seminal work, “Plantation Medicine,” once in “Medicine in the Confederacy,” and seven times in “Nurse Heroines of the Confederacy.” In closer examination for her works related to the Confederacy, a pattern emerges in phrasing associated with sentiments toward the Lost Cause myth. Both “Medicine in the Confederacy” and “Nurse Heroines of the Confederacy” mention the “cause” three and six times, respectively. In “Medicine in the Confederacy,” Marshall remarked, “without regard to the justice of their cause, no one can doubt the *whole-hearted conviction*, the *strength of purpose*, the *heroism* and the *sincerity* of the men and women of the South” [emphasis added] [79]. In one example, she described a surgeon's proud war efforts by “being the *leading spirit* in causing some three hundred southern medical students to withdraw from northern medical schools in 1860” [emphasis added] [80]. In another, she appealed to the strong conviction of the South as compared to the North during the American Civil War, writing “it is possible that this very disparity was a compelling force in demanding from the people of the South a *devotion to the cause* and a *unity of purpose* more widespread and more intense than was usual in the North” [emphasis added] [81]. As a prodigious orator and scholar, Marshall persuasively dismissed any debate on why the war was fought and instead sought agreement on terms that were hard to argue: the heroic characterization of those who fought with purpose. Persuasion was not needed due to her exclusively White audience and the place in which she spoke those words: Richmond, Virginia, the former capital of the Confederacy. In her three works, Marshall repeatedly used a phrase charismatically weaponized to promote the South's fight for state's rights while dismissing the truth that slavery was the main driver behind the American Civil War.

## DISCUSSION

“The belief that little on a subject has appeared in print, is to the reference worker as the proverbial gadfly, and so it has proved in this case.”—Mary Louise Marshall in “Medicine in the Confederacy”

Conducting critical librarianship in this field requires tackling the “white racial project” of librarianship and understanding how Whiteness, power, and privilege shape and impact practice, discourse, and legacies [82]. How else can the profession welcome voices within scholarly discourse without reconciling how Whiteness in the profession has historically excluded them? How should the profession make reparative efforts for the problematic scholarship published and disseminated in the profession's most prominent publication? Should the full scope of Marshall's scholarly record be known? Should a notation be assigned to Marshall's publications or listed on her MLA Fellows page? At present, none of her Southern medical works are listed as representative publications [83].

In addition, Marshall's works currently exist without any rebuttal of their controversial nature. No other articles were found that thoroughly examined Marshall's narratives nor contextualized Marshall's second abstention in withholding knowledge of the new integration policy. Lipscomb's documentation of the integration efforts missed critical analysis of Marshall's continued attempt to maintain a majority White organization. As a figure, Marshall was not only a leader in MLA for over a decade; she also penned influential works that shaped the profession. The narrative of her legacy predominantly focused on her achievements as president and practitioner. In some accounts, her three Southern works are incorrectly inflated as books rather than articles [84–86]. In this respect, she serves as one of the profession's cornerstones, and her formidable legacy acted as a barrier against representation then and acts as a problematic legacy now. Her works and ideologies expressed fear of comingling with Black and African American librarians and distinct notions on White racial superiority. It is imperative that her three works are recognized as examples of racism and not meeting the values of MLA. Health sciences librarians of different ethnicities need to be accepted as scholarly contributors, and the process in place to receive their works needs to be critically examined for institutionalized racism. Reflecting on this problematic past through acknowledgment of bias by the profession is one of several steps toward creating an inclusive scholarly community [87].

In 1989, Rachael K. Anderson delivered her Janet Doe Lecture on the recruitment of medical librarians and factors that hampered those efforts. In her speech, she hinted that the sentiments against integration were more widespread than Marshall alone:

Strong concerns were expressed in MLA that the attendance of blacks at annual meetings would create social problems and diminish the pleasure and value of these meetings for the rest of the membership … [t]he deeply-felt, negative, personal convictions of several individuals who were among the association's most active members and leaders for another generation betoken a continuing inhospitable climate for recruiting minorities to the field for many years thereafter [88].

It is not a stretch to believe that creating an “inhospitable climate” in retaining historically excluded librarians also influenced the absence of BIPOC voices in the scholarly discourse. By acknowledging the fact that the profession was not a welcoming space for BIPOC voices, it should be equally recognized that the support, tools, and knowledge needed to be involved within the profession, such as the practice of scholarly writing and research, were at best, difficult to procure, or at worst, nonexistent. Reckoning with this truth requires that journal editors, peer reviewers, and members of the profession make space and impart the tools necessary to encourage representation in the scholarly literature.

In addition, the Whiteness that permeates the pages of *BMLA* and *JMLA* in subsequent mentions of Marshall's legacy significantly whitewashes her contributions to the profession. Whether intentionally or unintentionally, this in turn omits, pivots, or downplays problematic issues of White figures in order to maintain their dignity and legacy.

Take for example a description of Marshall by John P. Isché in 1980:

For those youngsters who are not familiar with the name, let me present a brief resume: Emeritus Librarian and Emeritus Professor of Medical Bibliography of Tulane University School of Medicine … She has served on numerous committees and written extensively, including her chapter on classification in the first edition of the Hand book of Medical Library Practice and much more [89].

Isché went on to say, “members like this that made our association what it is” and concluded his commentary by stating, “we are proud of the contributions of our local MLAer—Mary Louise Marshall” [90]. For Isché, Marshall's achievements were symbolic of MLA's illustrious legacy. However, the works addressed in this study and her views against integration are glaringly absent. This incomplete picture, whether accidental or deliberate, shows how legacies become incorporated within the scholarly record and how they play a role in solidifying authority and professional norms. This should be recognized within all aspects of medical librarianship that are indoctrinated within the scholarly record. Are there other figures, practices, or recommendations that now require critical reexamination?

Lastly, Marshall's medical historical expertise and that as a librarian gave credibility to her works as authoritative topics for the benefit of other medical librarians and practitioners. As owner-by-proxy in the retelling of the enslaved narratives as described in “Plantation Medicine,” Marshall whitewashed and romanticized these topics while completely dismissing Black voices and experiences. Her perspective of Confederacy history in “Medicine in the Confederacy” and “Nurse Heroines in the Confederacy” and the use of her carefully crafted phrasing to describe the war is similar to others who found validity in the Lost Cause myth. How Marshall deliberately framed her narratives in favor of Whiteness and her own thoughts against integration must all be considered in her sentiments on race and her impact within MLA. In aggregate, Marshall's explicit biases found both in her works and actions as a library leader depicted a person with reservations for the progress of Black libraries and librarians while unequivocally supporting the professional advancement of White libraries and librarians. Whiteness allows for these biases to remain embedded in the profession, grossly affecting retention, representation, and invitations to publish, serve on conference panels, collaborate on research, and conduct peer review. It is imperative that members remain cognizant of these realities to combat biases as they appear within the association.

## CONCLUSION

This paper served to contextualize Marshall the library leader and practitioner with her ideology and how this influenced representation within MLA. In using Historical Revisionism theory, Whiteness as Property in CRT, and Whiteness in LIS theory, this study served to demonstrate how Marshall was a barrier for HBCU libraries and Black and African American librarians. In a similar vein, this work also examined the ways in which the profession minimized this important facet of medical librarianship history and how bringing awareness to this issue aims to move the profession forward. MLA is now at a critical moment in its existence. It is not possible to solely work from the present moment. In doing so, the profession fails to identify the historical apparatuses that have shaped the representation and progress of racially and ethnically diverse librarians in health sciences librarianship. Dismantling racism is how the profession will prosper now and into the future.

## STUDY LIMITATIONS

This study analyzed a small, important sampling of the works written by a prominent figure in the medical library profession. A critical approach was taken to examine these narratives; however, no author is free of bias, and depending on how Marshall's work is examined in the future, the author's subjectivity and perspective as a medical librarian does present its own limitations.

## AUTHOR NOTES

The author capitalized the proper nouns “Black” and “White” to center race and race dichotomy as important elements within the text. This was not changed for quoted text to keep with the authenticity of the primary source.

## Data Availability

Data associated with this article are available in the Open Science Framework at https://osf.io/cf2wq/files/.
